# Hybrid nanomaterials: from the laboratory to the market

**DOI:** 10.3762/bjnano.8.87

**Published:** 2017-04-13

**Authors:** Verónica de Zea Bermudez, Fabrice Leroux, Pierre Rabu, Andreas Taubert

**Affiliations:** 1 Department of Chemistry and CQ-VR, University of Trás-os-Montes e Alto Douro, 5000-801 Vila Real, Portugal; 2 Institute of Chemistry, ICCF – UMR CNRS 6296, University Clermont Auvergne, 24 av. des Landais, BP 80026, Aubière 63171, France; 3 Université de Strasbourg, CNRS, Institut de Physique et Chimie de Matériaux de Strasbourg, F-67034 Strasbourg, France; 4 Institute of Chemistry, University of Potsdam, D-14476 Potsdam, Germany

**Keywords:** hybrid nanomaterials

Materials, in all forms, are one of the mainstays of human development. The Stone Age would not have happened the same way without the ability of early humans to process stone into tools that could be used to their advantage. Similar observations can be made for every major step in human development up to the modern computer age where a whole zoo of different materials in very different amounts and very different forms is necessary to achieve the performance of countless devices that are ubiquitous today.

Along with active materials development by mankind, nature has developed highly complex, advanced materials with properties that are often superior to those made by humans. In this context, complexity is one of the keywords: biological materials are often highly complex, highly structured with well-defined hierarchy, with complex chemical compositions, often composed of a combination of organic and inorganic components, and therefore, they often have specifically adapted micro- and macroscopic properties and emergent phenomena. Materials from the natural world offer extraordinary features, such as self-healing, miniaturization, reconfigurability, and defect tolerance. Moreover, their synthesis typically takes place at roughly ambient conditions – often presenting a challenge to chemists, biologists, physicists, and engineers in its full complexity.

In spite of these challenges, we nowadays have a basic understanding of how (desired) material properties are related to material composition and structure. One of the key observations in this context is that the most desirable properties typically do not arise from a single material or chemical composition. Rather, these highly sought after properties, such as specific magnetic, optical, electrical, biological, or mechanical properties, arise from a delicate interplay of several material components of different origin, composition, and individual chemical and physical properties. As a result, some of the most attractive materials for modern technology are materials composed of at least two (and often more) different components – these are known as hybrid materials.

Indeed, biological high-performance materials such as bone or teeth are organic/inorganic hybrid materials of multiscale hierarchical structure and chemical composition perfectly matched to their respective task. As a result, hybrid materials have been explored for essentially all applications possible. Their chemical composition has been studied throughout the entire periodic table; countless organic molecules and polymers have been studied for their effects on structure, physical properties, and processability. Often, a “shotgun approach” towards the synthesis of new materials has been successful, and by serendipity, many interesting and useful materials have been found.

The recent past, however, has seen two developments that are key to the development and application of new, high-performance hybrid materials: (i) a new level of rational design has been reached − many molecular and supramolecular principles of hybrid material organization are not only known and quantified, but there are also strategies available to exploit these principles for materials design and materials production and (ii) the rapid development of computational and materials analysis tools, which provide much better insight into materials structure and properties than what was possible 15 or 20 years ago. As a result, we now understand some aspects of the interplay between chemical and physical properties much better, for example, between the chemical components and the macroscopic behavior of these materials. Consequently, it is also possible to make materials that have vastly improved properties by combination of carefully selected components with well-known individual properties.

As a result, there are many advanced hybrid materials available, some on a fairly large scale. Nevertheless, one of the remaining challenges is the transfer of these fascinating materials into large-scale applications in fields that are relevant to our modern society. Examples are healthcare, energy, environment, safety and security, but also less obvious are fields like arts and restoration. This clearly shows that, beyond developing new hybrid materials, there is a need to bring the most promising materials to the market for the benefit of the entire world.

This Thematic Series “Hybrid nanomaterials: from the laboratory to the market” was inspired by a series of symposia on advanced hybrid materials held at the E-MRS Spring Meetings in 2010, 2012, 2014, and 2016. From the beginning, the goal of the symposia was to foster discussion and exchange between groups active in this exciting yet very challenging field. The Thematic Series presents highlights from virtually all subfields of hybrid materials research. As the guest editors, we invite you to browse the issue, find among the many creative ideas presented here the ones that are most appealing to you, and to develop your own strong program in research, development, and application of advanced hybrid materials.

We would also like to thank the staff at the *Beilstein Journal of Nanotechnology*, along with all contributors and referees for making this exciting Thematic Series possible.

Verónica de Zea Bermudez, Fabrice Leroux, Pierre Rabu, and Andreas Taubert

Vila Real, Aubière, Strasbourg and Potsdam, March 2017

